# Differentiation of malignant and benign breast lesions: Added value of the qualitative analysis of breast lesions on diffusion-weighted imaging (DWI) using readout-segmented echo-planar imaging at 3.0 T

**DOI:** 10.1371/journal.pone.0174681

**Published:** 2017-03-30

**Authors:** Yeong Yi An, Sung Hun Kim, Bong Joo Kang

**Affiliations:** 1 Department of Radiology, St. Vincent’s Hospital, College of Medicine, The Catholic University of Korea, 93, Jungbu-daero, Paldal-gu, Suwon-si, Gyeonggi-do, Republic of Korea; 2 Department of Radiology, Seoul St. Mary’s Hospital, College of Medicine, The Catholic University of Korea, 222, Banpo-daero, Seocho-gu, Seoul, Republic of Korea; Norges teknisk-naturvitenskapelige universitet, NORWAY

## Abstract

**Objective:**

To determine the added value of qualitative analysis as an adjunct to quantitative analysis for the discrimination of benign and malignant lesions in patients with breast cancer using diffusion-weighted imaging (DWI) with readout-segmented echo-planar imaging (rs-EPI).

**Methods:**

A total of 99 patients with 144 lesions were reviewed from our prospectively collected database. DWI data were obtained using rs-EPI acquired at 3.0 T. The diagnostic performances of DWI in the qualitative, quantitative, and combination analyses were compared with that of dynamic contrast-enhanced magnetic resonance imaging (DCE-MRI). Additionally, the effect of lesion size on the diagnostic performance of the DWI combination analysis was evaluated.

**Results:**

The strongest indicators of malignancy on DWI were a heterogeneous pattern (P = 0.005) and an apparent diffusion coefficient (ADC) value <1.0 × 10–3 mm2/sec (P = 0.002). The area under the curve (AUC) values for the qualitative analysis, quantitative analysis, and combination analysis on DWI were 0.732 (95% CI, 0.651–0.803), 0.780 (95% CI, 0.703–0.846), and 0.826 (95% CI, 0.754–0.885), respectively (P<0.0001). The AUC for the combination analysis on DWI was superior to that for DCE-MRI alone (0.651, P = 0.003) but inferior to that for DCE-MRI plus the ADC value (0.883, P = 0.03). For the DWI combination analysis, the sensitivity was significantly lower in the size ≤1 cm group than in the size >1 cm group (80% vs. 95.6%, P = 0.034).

**Conclusions:**

Qualitative analysis of tumor morphology was diagnostically applicable on DWI using rs-EPI. This qualitative analysis adds value to quantitative analyses for lesion characterization in patients with breast cancer.

## Introduction

Dynamic contrast-enhanced magnetic resonance imaging (DCE-MRI) is widely used in breast cancer diagnosis and staging. DCE-MRI has widely demonstrated diagnostic value in breast imaging [1]. It provides high-resolution morphological information regarding the contrast-enhanced characteristics of the lesions. Although DCE-MRI has a high sensitivity of 94–100%, the specificity is only 40–80% for the characterization of the breast [2–4]. In addition to this relatively low specificity, DCE-MRI is time consuming and expensive. Furthermore, it carries the risk of potential side effects from the contrast media. Therefore, non-contrast-enhanced imaging techniques have been actively investigated as alternatives or adjuncts to DCE-MRI to detect breast cancer [4–8].

Diffusion-weighted MRI (DWI) is a promising non-contrast-enhanced imaging technique that is now well established in clinical breast MRI. DWI provides microstructural information regarding the diffusion of water molecules in the tissue cellularity and tissue structure. By using quantitative analysis with the apparent diffusion coefficient (ADC) values, the discrimination of benign and malignant breast lesions is possible, as well as the early identification of the treatment response in the neoadjuvant setting of breast cancer [9–14]. Although DWI can be interpreted both qualitatively and quantitatively, previous studies have primarily focused on the ADC quantification of breast lesions because of the reduced spatial resolution of DWI. There are few studies that have explored the use of this method for the qualitative morphological evaluation of breast lesions on DWI [15–16].

The most commonly used sequence for clinical DWI is the single-shot echo-planar imaging (ss-EPI) due to the tolerance to motion and short imaging time. However, the ss-EPI technique is prone to susceptibility artifacts, such as geometric distortion, signal dropout and image blurring [17]. To date, various advanced MR techniques have been proposed for distortion correction in EPI imaging [18–19]. The readout-segmented EPI (rs-EPI) is an alternative to ss-EPI for DWI with reduced distortion. The rs-EPI technique produces shorter echo-spacing than ss-EPI by dividing the k-space into separate segments in the readout direction [20]. The susceptibility artifact and image distortion can be reduced when rs-EPI is combined with parallel imaging [21]. In DWI of the breasts, previous studies have reported that rs-EPI images were of significantly higher image quality, spatial resolution and higher diagnostic accuracy than conventional ss-EPI [20–22]. In a previous work, we also demonstrated that rs-EPI was qualitatively superior to ss-EPI in terms of overall image quality, anatomical structure distinction and conspicuity of the lesions [23].

We hypothesized that DWI images obtained with rs-EPI would allow the morphological analysis of breast tumors and that the morphological information obtained via DWI could be used for breast lesion characterization, similar to the BI-RADS lexicon of DCE-MRI. Therefore, the aim of this study was to determine the diagnostic utility of qualitative analysis to assess the morphological features of breast lesions using DWI images with rs-EPI and to evaluate the added value for differentiation of malignant and benign lesions in patients with breast cancer.

## Materials and methods

### Patients

This study was approved by the Catholic Medical Center Office of Human Research Protection Program (CMC-OHRP)/Institutional Review Board (Approval No. KC13EISI0736), and all participants provided written informed consent for participation and publication of the research findings. Between November 2013 and November 2014, 99 patients (age range 33–83; mean age 55.4 years) with 144 pathologically verified lesions were enrolled (malignancy 112; high risk lesion 13; and benign lesion 19). The inclusion criteria were as follows: (1) no contraindications for the use of contrast agents, (2) no previous neoadjuvant chemotherapy or radiotherapy, and (3) BI-RADS 4/5 lesions detected on breast MRI with subsequent tissue confirmation (targeted ultrasound and ultrasound-guided biopsy or excision). The histopathological results are described in Table 1.

**Table 1:** Histopathological results.

Pathology	N (%)
Malignant	112 (77.8)
Invasive ductal carcinoma	78 (54.2)
Invasive lobular carcinoma	5 (3.5)
Mucinous carcinoma	2 (1.4)
Medullary carcinoma	1 (0.7)
Tubular carcinoma	3 (2.1)
Papillary carcinoma	2 (1.4)
Metaplastic carcinoma	3 (2.1)
Mucinous and papillary carcinoma	1 (0.7)
Ductal carcinoma in sity	15 (10.4)
Other	2 (1.4)
Benign	32 (22.2)
Fibrocystic change	14 (9.7)
Fibroadenoma	1 (0.7)
Stromal fibrosis	3 (2.1)
Histiocytic reaction	1 (0.7)
Sclerosing adenosis	2 (1.4)
Atypical ductal hyperplasia	4 (2.8)
Papilloma	5 (3.5)
Radial scar	1 (0.7)
Atypical ductal hyperplasia with papilloma	1 (0.7)

### Image acquisition

MRI images were acquired with the patient in the prone position using a 3.0 T scanner (Magnetom Verio; Siemens Medical Solutions, Erlangen, Germany) equipped with a breast coil. The following sequences were used: (1) axial, turbo spin-echo T2-weighted imaging sequence (TR/TE of 3530/93; flip angle 80°; 34 slices; FOV 320 × 320 mm; 576 × 403 matrix; 1 NEX; slice thickness 4 mm; and acquisition time 2 minutes 28 seconds); (2) axial DWI using rs-EPI (RESOLVE) (b = 0 and 750 seconds/mm^2^) (TR/TE 5600/55; echo train 32; spectral fat saturation (CHESS); phase encoding direction AP; voxel size 2.1 × 1.8 × 4.0 mm^3^; average 1; FOV 360 × 180 mm; matrix 192 × 82; slice thickness 4 mm; acquisition time 2 minutes 31 seconds; and 5 readout segments) and automatically generated ADC maps using built-in MRI software; and (3) pre- and post-contrast, axial T1-weighted flash 3D VIBE sequence [TR/TE of 4.4/1.7 ms; flip angle 10°; FOV 320 × 320 mm; 512 × 292 matrix; slice thickness 1.2 mm without interslice gap; slices per slab = 144; acquisition time 6 minutes 7 seconds; and obtained before and 7, 67, 127, 187, 247, and 307 seconds after an injection of 0.1 mmol/kg body weight of gadobutrol (Gadovist; Bayer Healthcare, Berlin, Germany)].

### Image analysis

#### DCE-MRI image analysis as the reference standard

All MRI images were reviewed by two breast radiologists with 13 and 6 years of experience in breast imaging. Any discrepancies were resolved by consensus. Morphological and kinetic analyses were performed on DCE-MRI using the American College of Radiology (ACR) Breast Imaging Reporting and Data System (BI-RADS) lexicon, fifth edition [24]. BI-RADS categories 1 to 3 were considered negative, while categories 4 and 5 were considered positive for malignancy.

#### Qualitative DWI analysis

**Fig 1:**
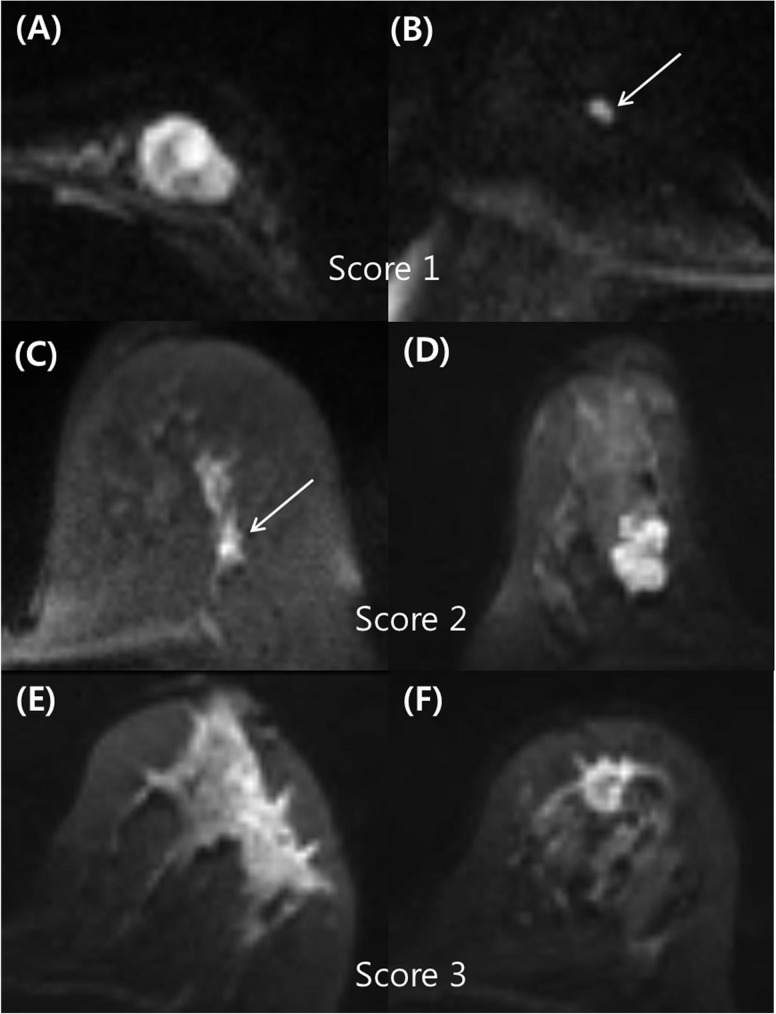
Examples of typical cases for each type of score. (A) A 43-year-old woman with a mucinous carcinoma in the left breast. The rs-EPI DWI demonstrated an oval circumscribed mass with a heterogeneous internal pattern in the left breast, which was scored as 1 (probably benign finding) during qualitative analysis. (B) An 80-year-old woman with invasive ductal carcinoma in the right breast. The rs-EPI DWI demonstrated an oval circumscribed mass with a homogeneous internal pattern in the right breast, which was scored a 1 (probably benign finding) on qualitative analysis. (C) A 61-year-old woman with ductal carcinoma in situ of the left breast. During qualitative analysis of rs-EPI DWI, there was a focal heterogeneous non-mass lesion, which was scored a 2 (indeterminate finding). (D) A 44-year-old woman with invasive ductal carcinoma in the right breast. The rs-EPI DWI demonstrated an irregular mass with an irregular margin and a homogeneous internal pattern, which was scored a 2 (indeterminate finding). (E) A 59-year-old woman with invasive ductal carcinoma of the left breast. During qualitative analysis of rs-EPI DWI, there was a segmental heterogenous non-mass that was scored a 3 (probably malignant finding). (F) A 51-year-old woman with invasive ductal carcinoma of the left breast. During qualitative analysis of rs-EPI DWI, there was an irregular spiculated mass with rim sign, which was scored a 3 (probably malignant finding).

If a hyperintense lesion was visible on index DWI images, the characteristics were subjected to qualitative DWI analysis using the morphological descriptors shown in Table 2. In contrast, if the lesion was not visible in the DW images, it was considered a negative case and then excluded from DWI analyses. The morphological descriptors for breast lesions used in the qualitative analysis on DWI are described in Table 2. The lesions were assessed with a three-level confidence score for qualitative analysis. These scores were characterized as follows: 1, probably benign (low probability of malignancy <10%); 2, indeterminate (intermediate probability of malignancy ranging from 10–50%); and 3, probably malignant (high probability of malignancy >50%). If the mass type lesions presented with minor findings associated with/without one intermediate descriptor or the non-mass type lesions presented with minor findings, they were scored a 1 (e.g., oval circumscribed mass with homogeneous/heterogeneous internal pattern). The mass type lesions with at least two intermediate findings or the non-mass type lesions with one intermediate finding were scored a 2 (e.g., non-circumscribed mass/non-mass lesion with heterogeneous internal pattern). Lesions with at least one major finding were scored a 3 (e.g., spiculated mass/segmental non-mass or non-circumscribed mass/non-mass with rim sign). A score of 1 was considered negative. Scores of 2 and 3 were classified as positive for malignancy. Examples of typical cases for each type of score are shown in Fig 1.

**Table 2:** Morphological descriptors used in DWI qualitative analysis according to estimated malignancy risk.

Lesion type		Minor	Intermediate	Major
Mass	Shape	Oval	Round	
			Irregular	
	Margin	Circumscribed	Irregular	Spiculated
Non-mass	Distribution	Focal	Linear	Segmental
Both	Internal pattern	Homogenous	Heterogenous	Rim sign

#### Quantitative DWI analysis

The ADC value was calculated according to the formula: ADC = [1/(b_2_-b_1_)]ln(S_2_/S_1_), where S_1_ and S_2_ are the signal intensities in the regions of interest (ROIs) obtained by two gradient factors, b_2_ and b_1_ (b_1_ = 0 and b_2_ = 750 seconds/mm^2^). A region of interest was manually drawn to encompass the corresponding lesion on the ADC maps. The DCE-MRI images were referenced to avoid fatty or necrotic tissues. At least three measurements were performed for each lesion. The lowest value was accepted as the ADC value. Receiver operating characteristic (ROC) curve analysis was performed to determine the optimal ADC cutoff value to differentiate malignant and benign tumors. The optimal cutoff value was determined by using the maximum Youden index (sensitivity + specificity– 1), assuming sensitivity and specificity were equally important. The resulting ADC cutoff value (1.0×10^-3^mm^2^/s) was used for the combination analysis in the next step.

#### Combination DWI analysis

We reassessed cases to evaluate the added value of qualitative analysis to quantitative analysis on DWI. For lesions that received a score of 1 or 2 on qualitative analysis, the final classification was based on the ADC value regardless of the qualitative score. All lesions with a score of 3 were classified as malignant regardless of the ADC value.

### Data and statistical analysis

To identify lesion characteristics in DWI analyses to predict malignancy, univariate and multivariate logistic regression analysis were performed by using the Chi-square test and Wilcoxon rank sum test (Table 3). Based on the univariate analysis results, we selected covariates for multivariate logistic regression analyses among the variables. These covariates were shape, margin, internal pattern, and ADC value for mass type lesions. We then calculated the odds ratios with 95% confidence intervals and P-values for each predictor variable. The diagnostic performance of each DWI analysis (qualitative alone, quantitative alone, and the combination of both qualitative and quantitative) was evaluated and compared with that of DCE-MRI by comparing ROC curve analyses. Following this, the diagnostic performance of the DWI combination analysis according to lesion size was evaluated using a Chi-square test and Hanley JA & McNeil BJ's method to determine the difference between two independent AUCs. All the statistical analyses were performed using the software package SAS Enterprise Guide 5.1 (SAS Institute, Inc., Cary, NC, USA) and MedCalc ver. 16.1 (MedCalc software, Mariakerke, Belgium). P-values <0.05 indicated statistical significance.

**Table 3:** DWI characteristics of 141 lesions for predicting malignancy.

	Total	Benign	Malignant	*p*-value‡
Lesion size				
mean (SD)	2.11±1.77	1.03±1.21	2.42±1.78	<0.0001
median(min-max)	1.7 (0.3–10.2)	0.75 (0.3–6.8)	2.0 (0.5–10.2)	<0.0001
≤1 cm	45 (31.5)	25 (55.6)	20 (44.4)	
>1 cm	98 (68.5)	7 (7.1)	91 (92.9)	
Lesion type				<0.0001
not seen	3 (2.1)	3 (100.0)	0 (0.0)	
mass	119 (82.6)	22 (18.5)	97 (81.5)	
nonmass	22 (15.3)	7 (31.8)	15 (68.2)	
Shape (for mass)				<0.0001
oval	23 (19.3)	12 (52.2)	11 (47.8)	
round	5 (4.2)	1 (20.0)	4 (80.0)	
irregular	91 (76.5)	10 (11.0)	81 (89.0)	
Margin (for mass)				<0.0001
circumscribed	15 (12.6)	9 (60.0)	6 (40.0)	
irregular	87 (73.1)	14 (16.1)	73 (83.9)	
spiculated	17 (14.3)	0 (0.0)	17 (100.0)	
Distribution (for nonmass)				0.0014
focal	10 (45.5)	7 (70.0)	3 (30.0)	
linear	2 (9.0)	0 (0.0)	2 (100.0)	
segmental	10 (45.5)	0 (0.0)	10 (100.0)	
Internal pattern (for both)				<0.0001
homogenous	39 (27.7)	21 (53.9)	18 (46.1)	
heterogenous	77 (54.6)	8 (10.4)	69 (89.6)	
rim sign	25 (17.7)	0 (0.0)	25 (100)	
ADC, ×10−^3^ mm^2^/s				<0.0001
mean (sd)	0.94±0.22	1.14±0.23	0.88±0.19	
median (min-max)	0.91(0.42–1.58)	1.15(0.66–1.58)	0.87(0.42–1.36)	
ADC, cutoff†				<0.0001
<1.0×10^-3^mm^2^/s	92 (65.25)	6 (6.5)	86 (93.5)	
≥1.0×10^-3^mm^2^/s	49 (34.75)	23 (46.9)	26 (53.1)	

Note_Values are presented as numbers (percentages) for categorical variables and mean (SD) and median (min-max) for continuous variables.

†The cutoff point determined by ROC curve with maximum Youden index.

‡P-values were calculated by using Chi-square test and Wilcoxon rank sum test.

## Results

The mean size of the malignant masses was 2.42 ± 1.78 cm, and the mean size of the benign masses was 1.03 ± 1.21 cm. The median sizes of the malignant and benign masses were 2.0 cm (range: 0.5–10.2 cm) and 0.75 cm (range: 0.3–6.8 cm), respectively. Three lesions were not visible on DWI: a 1.5-cm radial scar, a 0.6-cm intraductal papilloma, and a fibrocystic change of 0.4 cm. Of the 141 lesions visible on DWI, 119 were mass type lesions, and 22 were non-mass type lesions.

**Fig 2:**
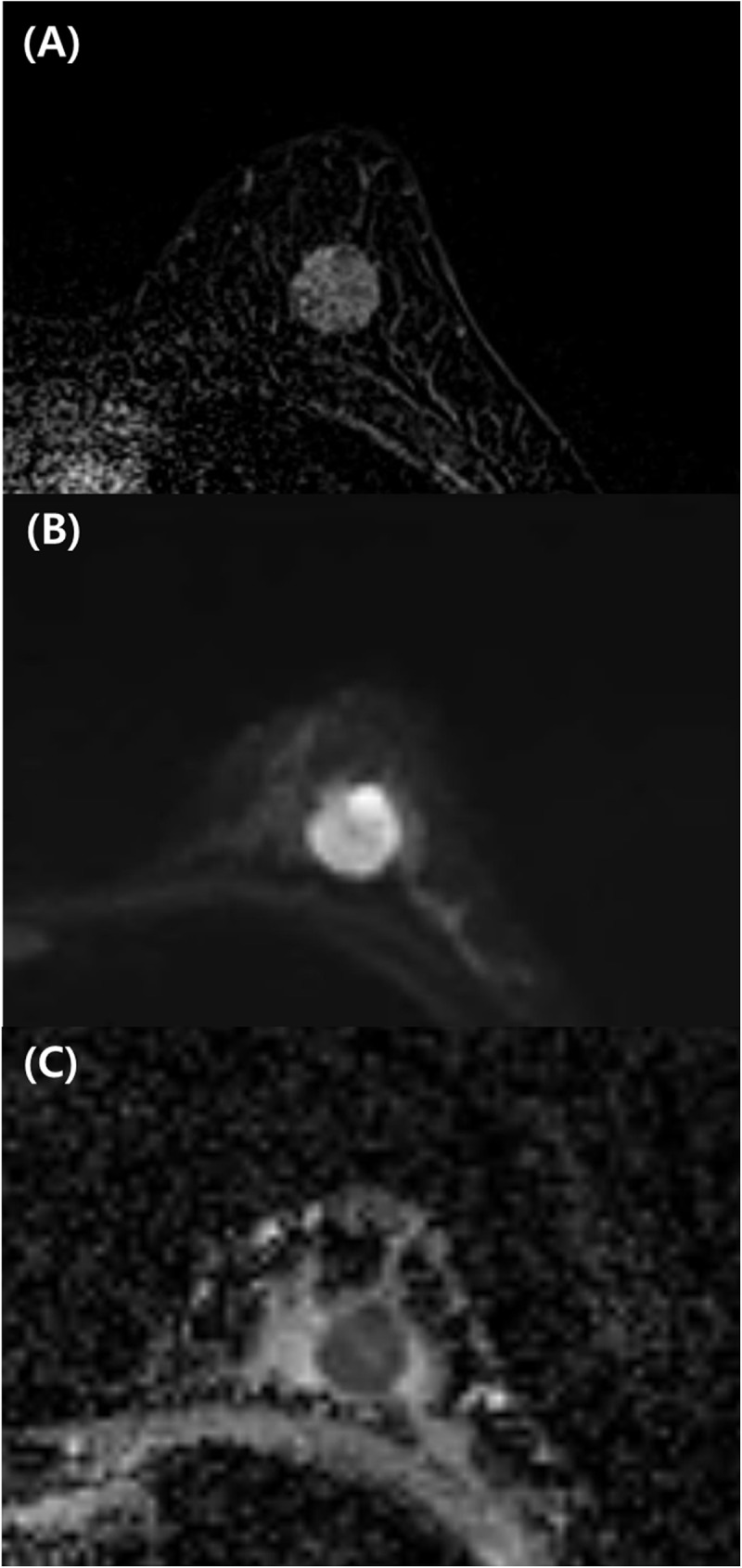
33-year-old woman with invasive ductal carcinoma in the left breast. Contrast-enhanced T1-weighted axial image (A), readout-segmented echo-planar DWI image (B), and ADC map (C). (A) Round circumscribed mass with heterogeneous enhancement in the breast. With DWI, at 750 seconds/mm^2^, there is a round circumscribed mass with heterogeneous high signal intensity in the left breast (B) with low ADC (0.8×10−^3^ mm^2^/sec) (C). The patient underwent breast-conserving surgery. The final diagnosis was invasive ductal carcinoma of histological grade III and triple-negative subtype.

The significantly frequent morphological features among the malignancies were round/irregular shapes and irregular/spiculated margins for mass type lesions (P<0.0001, Table 3). For non-mass type malignancies, the significant features were linear/segmental distribution and heterogeneous internal pattern/rim sign (P<0.0001, Table 3). The significantly frequent morphological features among benign breast lesions were an oval shape and a circumscribed margin for mass type lesions (P<0.0001) and focal and homogeneous internal patterns for non-mass type lesions (P<0.0001). For univariate analysis, the following DWI features were significantly different between benign and malignant lesions: shape, margin, the pattern, and ADC cutoff value of 1.0×10−^3^ mm^2^/s for mass type lesions. Lesion characteristics for NME type lesions were not included in the multivariate analysis because the variables were not significant in the univariate analysis. With multivariate logistic regression analyses (Table 4), a heterogeneous internal pattern (P = 0.005) and ADC value <1.0×10^-3^mm^2^/s (P = 0.002) were the DWI features with the strongest independent indications for malignancy (Fig 2).

**Table 4:** Univariate and multivariate logistic regression analysis of DWI characteristics for predicting malignancy.

Lesion characteristics (for mass type)	No. of benign lesions (%)	No. of malignant lesions (%)	Univariate analysis		Multivariate analysis	
			Odds Ratio (95% CI)	*p*-value†	Odds Ratio (95% CI)	*p*-value‡
Qualitative DWI analysis	25 (20.7)	96 (79.3)				
Shape						
oval	14 (53.9)	12 (46.2)	1		1	
round	2 (33.3)	4 (66.7)	2.33 (0.36–15.05)	0.373	1.33 (0.05–37.46)	0.869
irregular	9 (10.1)	80 (89.9)	10.37 (3.69–29.16)	<0.0001	13.56 (0.64–289.51)	0.095
Margin						
circumscribed	13 (54.2)	11 (45.8)	1		1	
irregular	12 (16.7)	60 (83.3)	5.91 (2.14–16.29)	0.0006	0.32 (0.01–10.39)	0.523
spiculated	0 (0)	25 (100.0)	…	0.942	…	0.966
Internal pattern						
homogenous	19 (82.6)	4 (17.4)	1		1	
heterogenous	6 (9.38)	58 (90.6)	45.92 (11.70–180.18)	<0.0001	21.34 (2.44–186.81)	0.006
rim sign	0 (0)	34 (100.0)	…	0.926	…	0.947
Quantitative DWI analysis	22 (18.6)	96 (81.4)				
ADC<1.0×10^-3^mm^2^/s	5 (5.9)	79 (94.1)	15.80 (5.12–48.74)	<0.0001	19.07 (2.79–130.24)	0.003
ADC≥1.0×10^-3^mm^2^/s	17 (50.0)	17 (50.0)	1			

Note_ Lesion characteristics for NME type lesions were not included in the multivariate analysis because variables were not significant in the univariate analysis.

†Determined with the χ^2^ test.

‡Determined with logistic regression analysis.

Table 5 summarizes the distributions of scores for the qualitative DWI analysis and BI-RADS categories of the morphological analysis of DCE-MRI. For the combined DWI analysis, 7 of 21 lesions with score 1 on the qualitative DWI analysis demonstrated an ADC value<1.0×10−^3^ mm^2^/s and were upgraded to score 2. Then, 15 of 32 lesions with score 2 on the qualitative DWI analysis demonstrated ADC≥1.0×10^-3^mm^2^/s and were downgraded to score 1. For the lesions with score 3, on the qualitative DWI analysis, 22.73% had ADC≥1.0×10^-3^mm^2^/s. When DCE-MRI analysis was combined with the ADC value, 3 of 12 lesions with BI-RADS category 3 were upgraded to BI-RADS category 4, and 20 of 28 lesions with BI-RADS category 4 were downgraded to BI-RADS 3 category. For the lesions with BI-RADS category 5, 22.11% had ADC≥1.0×10^-3^mm^2^/s.

**Table 5:** Distributions of scores for the qualitative DWI analysis and BI-RADS categories of the morphological analysis of DCE-MRI.

	No. of ADC<1.0×10−^3^ mm^2^/s (%)	No. of ADC≥1.0×10−^3^ mm^2^/s (%)	Total
DWI, qualitative†			
Score 1	7 (33.30)	14 (66.70)	21
Score 2	17 (53.12)	15 (48.88)	32
Score 3	68 (77.27)	20 (22.73)	88
DCE-MRI, morphology‡			
BI-RADS 3	3 (25.00)	9 (75.00)	12
BI-RADS 4	8 (28.57)	20 (71.43)	28
BI-RADS 5	81 (77.88)	23 (22.11)	104

†n = 141

‡n = 144.

**Fig 3:**
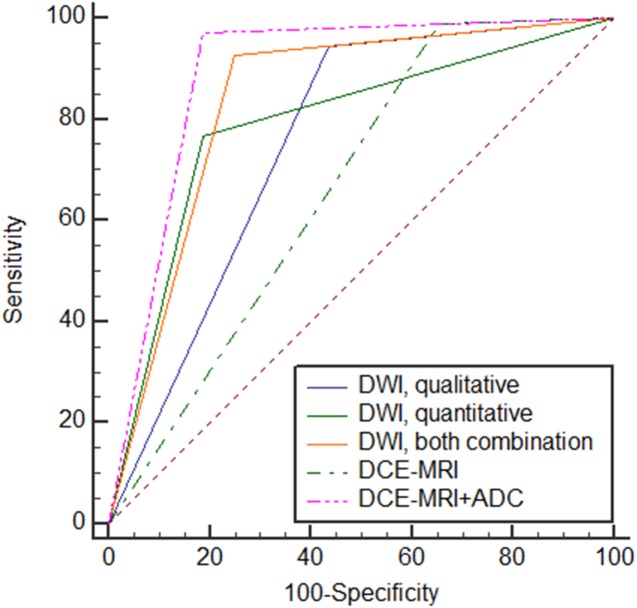
ROC analysis comparing the diagnostic performances of DWI (qualitative alone, quantitative alone, and combination) and DCE-MRI (DCE-MRI alone, DCE-MRI plus ADC).

ROC analysis comparing the diagnostic performances of DWI and DCE-MRI is described in Table 6 and Fig 3. The AUCs of (1) qualitative DWI analysis only, (2) quantitative DWI analysis only, (3) combined DWI analysis, (4) DCE-MRI alone, and (5) DCE-MRI plus ADC were 0.732 (95% CI, 0.651–0.803), 0.780 (95% CI, 0.703–0.846), 0.826 (95% CI, 0.754–0.885), 0.651 (95% CI, 0.566–0.729), and 0.883 (95% CI, 0.818–0.931), respectively (Fig 3). The AUCs of the independent qualitative and quantitative DWI analyses were lower than the AUC of the DWI combination analysis, although the difference was not statistically significant. The AUC of the DWI combination analysis was superior to that of DCE-MRI only but was inferior to that of DCE-MRI plus ADC value (P = 0.003 and P = 0.03, respectively).

**Table 6:** Comparing the diagnostic performances of DWI and DCE-MRI.

	DWI, qualitative†	DWI, quantitative†	DWI, combination†	DCE-MRI only‡	DCE-MRI+ADC‡
Sensitivity (%)	94.64	76.79	92.86	99.11	97.32
Specificity (%)	51.72	79.31	72.41	34.38	81.25
PPV^a^ (%)	88.33	93.48	92.86	84.09	94.78
NPV^b^ (%)	71.43	46.94	72.41	91.67	89.66
Accuracy (%)	85.80	77.30	88.65	84.72	93.75
AUC^c^ (95% CI^d^)	0.732 (0.651–0.803)	0.780 (0.703–0.846)	0.826 (0.754–0.885)	0.651 (0.566–0.729)	0.883 (0.818–0.931)

^a^PPV, positive predictive value

^b^NPV, negative predictive value

^c^AUC, area under the curve

^d^CI, confidence interval

†n = 141

‡n = 144

The diagnostic performance of the DWI combination analysis according to lesion size is shown in Table 7. The sensitivity and positive predictive value of the size ≤1 cm group (80% and 72.7%, respectively) were significantly lower than those of the size >1 cm group (95.6% and 97.8%, respectively) (P = 0.034, P = <0.0001). However, the other diagnostic indices did not differ significantly according to lesion size.

**Table 7:** Diagnostic performance of the DWI combination analysis by lesion size.

	MR size ≤1 cm	MR size >1 cm	*p*-value†
Sensitivity (%)	80.00	95.60	0.0340
Specificity (%)	76.00	71.43	>0.9999
PPV^a^ (%)	72.73	97.75	<0.0001
NPV^b^ (%)	82.61	55.56	0.0745
Accuracy (%)	77.78	93.88	0.0745
AUC^c^ (95% CI^d^)	0.78	0.84	0.6231

^a^PPV, positive predictive value

^b^NPV, negative predictive value

^c^AUC, area under the curve

^d^CI, confidence interval.

†*p*-values were calculated using a Chi-square test and Hanley JA & McNeil BJ's method to determine the difference between two independent AUCs.

## Discussion

Our study demonstrated that qualitative DWI analysis based on morphological analysis was useful in predicting malignancy and has a potential to improve the diagnostic performance of DWI. Using multivariate analysis, the heterogeneous internal pattern of various morphological descriptor on DWI, such as that of a low ADC value (<1.0×10−^3^ mm^2^/sec), was the most significant independent predictor of malignancy. Additionally, the combined DWI analysis enabled improved diagnostics to predict breast cancer by increasing sensitivity without a loss of specificity in quantitative ADC analysis, although it was inferior to the combination of DCE-MRI and ADC. Currently, there have been few studies that evaluate the diagnostic usefulness of the morphological analysis of breast lesions on DWI [15–16]. Previously, Kang et al. investigated the diagnostic accuracy and usefulness of a high signal rim sign on DWI [15]. The sensitivities, specificities, and AUC values were 59.7%, 80.6%, and 0.701, respectively, for the rim sign and 82.3%, 63.9%, and 0.731, respectively, for the ADC value (cutoff ≤1.46×10−^3^ mm^2^/sec). Their results suggested that a high signal rim sign on DWI was a valuable morphological feature to improve specificity in DWI. However, they only focused on one morphological characteristic on DWI, the rim sign. We think that further evaluation of the diagnostic performance and positive predictive values of each morphological descriptor are necessary for the differentiation between benign and malignant lesions on DWI. Recently, Barentsz et al. examined the diagnostic value of qualitative analysis of DWI using the reduced field-of-view (rFOV) technique in 30 breast lesions [16]. In that study, the shape and BI-RADS classification of the lesions were considered in the qualitative analysis. The discriminative abilities based on ADC values were similar for ss-EPI and rFOV, with AUCs of 0.79 and 0.82, respectively. When the lesion shape was included in the analysis, the AUCs from the three readers ranged from 0.74 to 0.91 for rFOV and from 0.67 to 0.75 for ss-EPI. When the BI-RADS classification of the lesion was added to the interpretation, the AUCs for the three readers were 0.71–0.93 for rFOV, 0.61–0.76 for ss-EPI, and 0.87–0.91 for DCE-MRI. These results suggested that additional assessment of tumor morphology with rFOV contributed to the higher AUCs, which is consistent with our results. However, the rFOV technique has two major limitations: unilateral breast coverage and additional scan time as an adjunct to standard DWI. Therefore, we believe that the rs-EPI technique is advantageous over rFOV because it can cover the entire breast.

Recently, breast DWI has been investigated as a single modality or in combination with other unenhanced MRI sequences. Combining DWI with other non-enhanced MR sequences may overcome the limitations by simultaneously providing anatomical and functional information about the breast tumors [25–29]. Our study focused on the diagnostic potential of DWI with rs-EPI alone. The lesion detection rate on DWI with rs-EPI was 97.9%. Two of the three invisible lesions on DWI were <1 cm. Based on the lesion size, the sensitivity and positive predictive value were significantly less in the size ≤1 cm group than in the size >1 cm group. Although the rs-EPI technique had higher diagnostic accuracy than ss-EPI in previous studies [20, 22–23], our results demonstrate that the spatial resolution of the rs-EPI technique at 3 T was limited in the detection of early breast cancer. The relatively low spatial resolution and distortion of DWI influenced lesion detectability. A small lesion below the spatial resolution of DWI may not be suitable for qualitative morphological analysis. In the lesion characterization, the marginal features of a small lesion (i.e., spiculated margin) could not be clearly demonstrated and thus could not contribute to the differential diagnosis between benign and malignant lesions. In a recently published study by Bogner et al. [21], DWI with combined parallel imaging and rs-EPI at 7 T yielded high-quality ADC maps and submillimeter in-plane high-spatial resolution images for lesion characterization. This reflects the potential of DWI of the breast at 7 T to overcome the former restrictions in spatial resolution. The morphological assessment of breast tumors with high-resolution DWI can play an important role in overcoming these limitations.

In this study, the morphological features of the breast tumors were visually assessed by radiologists, which is subjective to high intra- and interobserver variability. Textural analysis can describe the relation of the gray levels between neighboring pixels by applying various mathematical methods, and it can provide quantitative morphological features regarding tumor spatial heterogeneity, which has been known to be an important prognostic factor [30]. Intratumoral spatial information can be quantified as a range of parameters. Previous studies have investigated the clinical application of textural analysis for the differentiation of benign from malignant breast lesions in DCE-MRI [31–34]. Although the application in breast DW-MRI has yet to be widely investigated [35], we expect it has promising potential to provide objective and quantitative morphologic parameters of the breast lesions on DWI. However, the implementation of texture analysis is promising while remaining challenging in routine clinical use. The one-to-one correspondence relationship between the quantitative morphologic features of textural analysis and the morphological descriptors of BI-RADS lexicon have yet to be established [36–37]. In addition, the effects of image acquisition and image quality on textural analysis with MRI should also be explored in future investigations.

Our study had several limitations. First, the patients were selected from those that were assigned for preoperative analysis. Therefore, selection bias may have been introduced. Second, our study populations included mostly malignant lesions, with a relatively small number of benign lesions. This may make it difficult to generalize our results to the entire spectrum of breast lesions. Therefore, further investigations with a more widespread distribution of histopathological subtypes are necessary to corroborate the results of this study. Third, the morphological descriptors used in DWI qualitative analysis are arbitrary, and scoring can be subjective. Interobserver and intraobserver variability is also not considered for image interpretations because all images were interpreted by two radiologists in consensus. As briefly discussed above, textural analysis using computerized software could overcome the limitation of visual assessment and provide more objective quantitative morphological information.

In conclusion, DWI with the rs-EPI technique is both diagnostically applicable and useful for predicting malignancy. Multivariate analyses demonstrated that the best malignant predictors for DWI were a heterogeneous internal pattern and a low ADC value(<1.0×10^−3^ mm^2^/s). The combined DWI analysis improved the diagnostic performance of DWI to predict breast cancer by increasing sensitivity without a loss of specificity, although it was inferior to that of DCE-MRI plus ADC. DWI using the rs-EPI technique at 3 T still demonstrated limited spatial resolution for the detection of small breast lesions (<1 cm). Therefore, the development of a high-resolution DWI technique is needed. Further large prospective studies are needed to evaluate the contribution of high-resolution DWI as an adjunct or alternative MRI technique to DCE-MRI for the detection and characterization of breast cancer.

## Supporting information

S1 Dataset The basic dataset of this study. This is the basic dataset of this study including pathologic and radiologic information.
(XLSX)

## References

[1] DeMartini W, Lehman C, Partridge S. Breast MRI for cancer detection and characterization: a review of evidence-based clinical applications. Academic radiology. 2008;15(4):408–16. 10.1016/j.acra.2007.11.006 18342764

[2] Fischer U, Kopka L, Grabbe E. Breast carcinoma: effect of preoperative contrast-enhanced MR imaging on the therapeutic approach. Radiology. 1999;213(3):881–8. 10.1148/radiology.213.3.r99dc01881 10580970

[3] Orel SG, Schnall MD. MR imaging of the breast for the detection, diagnosis, and staging of breast cancer. Radiology. 2001;220(1):13–30. 10.1148/radiology.220.1.r01jl3113 11425968

[4] Park MJ, Cha ES, Kang BJ, Ihn YK, Baik JH. The role of diffusion-weighted imaging and the apparent diffusion coefficient (ADC) values for breast tumors. Korean journal of radiology. 2007;8(5):390–6. 10.3348/kjr.2007.8.5.390 17923781 PMC2626812

[5] Ballesio L, Savelli S, Angeletti M, Porfiri LM, D'Ambrosio I, Maggi C, et al Breast MRI: Are T2 IR sequences useful in the evaluation of breast lesions? European journal of radiology. 2009;71(1):96–101. 10.1016/j.ejrad.2008.03.025 18479866

[6] Malich A, Fischer DR, Wurdinger S, Boettcher J, Marx C, Facius M, et al Potential MRI interpretation model: differentiation of benign from malignant breast masses. AJR American journal of roentgenology. 2005;185(4):964–70. 10.2214/AJR.04.1073 16177416

[7] Partridge SC, Rahbar H, Murthy R, Chai X, Kurland BF, DeMartini WB, et al Improved diagnostic accuracy of breast MRI through combined apparent diffusion coefficients and dynamic contrast-enhanced kinetics. Magnetic resonance in medicine. 2011;65(6):1759–67. 10.1002/mrm.22762 21254208 PMC3201817

[8] Yuen S, Uematsu T, Kasami M, Tanaka K, Kimura K, Sanuki J, et al Breast carcinomas with strong high-signal intensity on T2-weighted MR images: pathological characteristics and differential diagnosis. Journal of magnetic resonance imaging: JMRI. 2007;25(3):502–10. 10.1002/jmri.20845 17326093

[9] Choi SY, Chang YW, Park HJ, Kim HJ, Hong SS, Seo DY. Correlation of the apparent diffusion coefficiency values on diffusion-weighted imaging with prognostic factors for breast cancer. The British journal of radiology. 2012;85(1016):e474–9. 10.1259/bjr/79381464 22128125 PMC3587081

[10] Jeh SK, Kim SH, Kim HS, Kang BJ, Jeong SH, Yim HW, et al Correlation of the apparent diffusion coefficient value and dynamic magnetic resonance imaging findings with prognostic factors in invasive ductal carcinoma. Journal of magnetic resonance imaging: JMRI. 2011;33(1):102–9. 10.1002/jmri.22400 21182127

[11] Marini C, Iacconi C, Giannelli M, Cilotti A, Moretti M, Bartolozzi C. Quantitative diffusion-weighted MR imaging in the differential diagnosis of breast lesion. European radiology. 2007;17(10):2646–55. 10.1007/s00330-007-0621-2 17356840

[12] Partridge SC, Mullins CD, Kurland BF, Allain MD, DeMartini WB, Eby PR, et al Apparent diffusion coefficient values for discriminating benign and malignant breast MRI lesions: effects of lesion type and size. AJR American journal of roentgenology. 2010;194(6):1664–73. 10.2214/AJR.09.3534 20489111

[13] Yabuuchi H, Matsuo Y, Okafuji T, Kamitani T, Soeda H, Setoguchi T, et al Enhanced mass on contrast-enhanced breast MR imaging: Lesion characterization using combination of dynamic contrast-enhanced and diffusion-weighted MR images. Journal of magnetic resonance imaging: JMRI. 2008;28(5):1157–65. 10.1002/jmri.21570 18972357

[14] Kul S, Cansu A, Alhan E, Dinc H, Gunes G, Reis A. Contribution of diffusion-weighted imaging to dynamic contrast-enhanced MRI in the characterization of breast tumors. AJR American journal of roentgenology. 2011;196(1):210–7. 10.2214/AJR.10.4258 21178069

[15] Kang BJ, Lipson JA, Planey KR, Zackrisson S, Ikeda DM, Kao J, et al Rim sign in breast lesions on diffusion-weighted magnetic resonance imaging: diagnostic accuracy and clinical usefulness. Journal of magnetic resonance imaging: JMRI. 2015;41(3):616–23. 10.1002/jmri.24617 24585455 PMC7674005

[16] Barentsz MW, Taviani V, Chang JM, Ikeda DM, Miyake KK, Banerjee S, et al Assessment of tumor morphology on diffusion-weighted (DWI) breast MRI: Diagnostic value of reduced field of view DWI. Journal of magnetic resonance imaging: JMRI. 2015;42(6):1656–65. 10.1002/jmri.24929 25914178 PMC4619182

[17] Holdsworth SJ, Yeom K, Skare S, Gentles AJ, Barnes PD, Bammer R. Clinical application of readout-segmented- echo-planar imaging for diffusion-weighted imaging in pediatric brain. AJNR American journal of neuroradiology. 2011;32(7):1274–9. 10.3174/ajnr.A2481 21596809 PMC3985839

[18] Teruel JR, Fjøsne HE, Østlie A, Holland D, Dale AM, Bathen TF, et al Inhomogeneous static magnetic field-induced distortion correction applied to diffusion weighted MRI of the breast at 3T. Magn Reson Med. 2015;74(4):1138–44. 10.1002/mrm.25489 25323982

[19] Hancu I, Lee SK, Hulsey K, Lenkinski R, Holland D, Sperl JI, et al Distortion correction in diffusion-weighted imaging of the breast: Performance assessment of prospective, retrospective, and combined (prospective+retrospective) approaches. Magn Reson Med. 2016 7 12. [Epub ahead of print] 10.1002/mrm.26328 PMC5233674 27403765

[20] Bogner W, Pinker-Domenig K, Bickel H, Chmelik M, Weber M, Helbich TH, et al Readout-segmented echo-planar imaging improves the diagnostic performance of diffusion-weighted MR breast examinations at 3.0 T. Radiology. 2012;263(1):64–76. 10.1148/radiol.12111494 22438442

[21] Bogner W, Pinker K, Zaric O, Baltzer P, Minarikova L, Porter D, et al Bilateral diffusion-weighted MR imaging of breast tumors with submillimeter resolution using readout-segmented echo-planar imaging at 7 T. Radiology. 2015;274(1):74–84. 10.1148/radiol.14132340 25341078

[22] Wisner DJ, Rogers N, Deshpande VS, Newitt DN, Laub GA, Porter DA, et al High-resolution diffusion-weighted imaging for the separation of benign from malignant BI-RADS 4/5 lesions found on breast MRI at 3T. J Magn Reson Imaging. 2014;40(3):674–81. 10.1002/jmri.24416 24214467 PMC4014534

[23] Kim YJ, Kim SH, Kang BJ, Park CS, Kim HS, Son YH, et al Readout-segmented echo-planar imaging in diffusion-weighted mr imaging in breast cancer: comparison with single-shot echo-planar imaging in image quality. Korean J Radiol. 2014;15(4):403–10. 10.3348/kjr.2014.15.4.403 25053898 PMC4105801

[24] American College of Radiology. Breast Imaging and Reporting and Data System (ACR BI-RADS Atlas), 5^th^ ed. Reston, VA: American College of Radiology, 2013.

[25] Baltzer PA, Benndorf M, Dietzel M, Gajda M, Camara O, Kaiser WA. Sensitivity and specificity of unenhanced MR mammography (DWI combined with T2-weighted TSE imaging, ueMRM) for the differentiation of mass lesions. European radiology. 2010;20(5):1101–10. 10.1007/s00330-009-1654-5 19936758

[26] Kuroki-Suzuki S, Kuroki Y, Nasu K, Nawano S, Moriyama N, Okazaki M. Detecting breast cancer with non-contrast MR imaging: combining diffusion-weighted and STIR imaging. Magn Reson Med Sci. 2007;6(1):21–7. 17510539 10.2463/mrms.6.21

[27] Trimboli RM, Verardi N, Cartia F, Carbonaro LA, Sardanelli F. Breast cancer detection using double reading of unenhanced MRI including T1-weighted, T2-weighted STIR, and diffusion-weighted imaging: a proof of concept study. AJR American journal of roentgenology. 2014;203(3):674–81. 10.2214/AJR.13.11816 25148175

[28] Tsushima Y, Takano A, Taketomi-Takahashi A, Endo K. Body diffusion-weighted MR imaging using high b-value for malignant tumor screening: usefulness and necessity of referring to T2-weighted images and creating fusion images. Academic radiology. 2007;14(6):643–50. 10.1016/j.acra.2007.02.006 17502253

[29] Yabuuchi H, Matsuo Y, Sunami S, Kamitani T, Kawanami S, Setoguchi T, et al Detection of non-palpable breast cancer in asymptomatic women by using unenhanced diffusion-weighted and T2-weighted MR imaging: comparison with mammography and dynamic contrast-enhanced MR imaging. European radiology. 2011;21(1):11–7. 10.1007/s00330-010-1890-8 20640898

[30] Davnall F, Yip CS, Ljungqvist G, Selmi M, Ng F, Sanghera B, et al Assessment of tumor heterogeneity: an emerging imaging tool for clinical practice? Insights Imaging. 2012 12;3(6):573–89. 10.1007/s13244-012-0196-6 23093486 PMC3505569

[31] Woods BJ, Clymer BD, Kurc T, Heverhagen JT, Stevens R, Orsdemir A, et al Malignant-lesion segmentation using 4D co-occurrence texture analysis applied to dynamic contrast-enhanced magnetic resonance breast image data. Journal of magnetic resonance imaging: JMRI. 2007 3;25(3):495–501. 10.1002/jmri.20837 17279534

[32] Chen W, Giger ML, Li H, Bick U, Newstead GM. Volumetric texture analysis of breast lesions on contrast-enhanced magnetic resonance images. Magn Reson Med. 2007 9;58(3):562–71. 10.1002/mrm.21347 17763361

[33] Karahaliou A, Vassiou K, Arikidis NS, Skiadopoulos S, Kanavou T, Costaridou L. Assessing heterogeneity of lesion enhancement kinetics in dynamic contrast-enhanced MRI for breast cancer diagnosis. Br J Radiol. 2010 4;83(988):296–309. 10.1259/bjr/50743919 20335440 PMC3473457

[34] Preim U, Glaßer S, Preim B, Fischbach F, Ricke J. Computer-aided diagnosis in breast DCE-MRI-quantification of the heterogeneity of breast lesions. Eur J Radiol. 2012 7;81(7):1532–8. 10.1016/j.ejrad.2011.04.045 21570225

[35] Yun BL, Cho N, Li M, Jang MH, Park SY, Kang HC, et al Intratumoral heterogeneity of breast cancer xenograft models: texture analysis of diffusion-weighted MR imaging. Korean J Radiol. 2014 Sep-Oct;15(5):591–604. 10.3348/kjr.2014.15.5.591 25246820 PMC4170160

[36] Nie K, Chen JH, Yu HJ, Chu Y, Nalcioglu O, Su MY. Quantitative analysis of lesion morphology and textural features for diagnostic prediction in breast MRI. Acad Radiol. 2008 12;15(12):1513–25. 10.1016/j.acra.2008.06.005 19000868 PMC2791407

[37] Newell D, Nie K, Chen JH, Hsu CC, Yu HJ, Nalcioglu O, Su MY. Selection of diagnostic features on breast MRI to differentiate between malignant and benign lesions using computer-aided diagnosis: differences in lesions presenting as mass and non-mass-like enhancement. Eur Radiol. 2010 4;20(4):771–81. 10.1007/s00330-009-1616-y 19789878 PMC2835636

